# Host–guest interaction of cucurbit[8]uril with oroxin A and its effect on the properties of oroxin A

**DOI:** 10.3762/bjoc.16.194

**Published:** 2020-09-22

**Authors:** Zhishu Zeng, Jun Xie, Guangyan Luo, Zhu Tao, Qianjun Zhang

**Affiliations:** 1 Key Laboratory of Macrocyclic and Supramolecular Chemistry of Guizhou Province, Guizhou University, No. 2708, South Section of Huaxi Avenue, Huaxi, Guiyang 550025, China

**Keywords:** cucurbit[8]uril, host–guest interaction, inclusion complex, oroxin A, properties

## Abstract

In this study, we investigated the host–guest interactions between oroxin A (OA) and cucurbit[8]uril (Q[8]) using ^1^H NMR, MS, UV–vis and IR spectroscopy. The results showed that OA and Q[8] formed an inclusion compound (OA@Q[8]) with a molar ratio of 1:1 and a binding constant of 1.299 × 10^7^ L·mol^−1^. In addition, the effect of Q[8] on the properties of OA was investigated through comparative experiments. The solubility of OA in water increased 22.47-fold when the concentration of Q[8] was 1 × 10^−4^ mol·L^−1^. Q[8] hardly affected the antioxidant capacity of OA, while the cumulative release of OA in gastric juice increased 2.3-fold after forming the inclusion compound with Q[8].

## Introduction

Cucurbit[*n*]urils (Q[*n*]s) are a family of macrocyclic cage compounds synthesized by the condensation of glycoluril and formaldehyde in a strong acidic solution [1–3]. As a consequence of the specific structural features of Q[*n*]s, which have two hydrophilic “portals” decorated with partially negatively charged carbonyl groups and a hydrophobic cavity [4], cucurbit[*n*]urils are able to form host–guest complexes with a range of drugs [5–7]. These complexes involve three main intermolecular forces: a hydrophobic effect, hydrogen bonding and ion–dipole interactions at the carbonyl portals [7–9]. The high thermal stability [10], ease of synthesis [11], general absence of cytotoxicity or toxicity [12–13] and their good molecular recognition and binding constants [14] have shown that Q[*n*]s are ideal drug carriers [15–16]. Moreover, Q[*n*]s can enhance the physical stability [17–18] and increase the solubility [19–20] of drug molecules. After Q[*n*]s forms a host–guest complex with drug molecules, it can also improve the bioavailability and delivery capacity [21], can help to reduce the side effects and toxicity of the drug [22].

**Figure 1:**
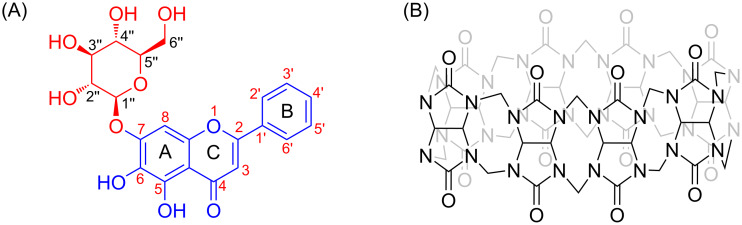
The molecular structure of OA (A) and Q[8] (B).

Oroxin A (OA, baicalein-7-O-glucoside, Figure 1A) is one of the active ingredients isolated from the traditional herbal medicine *Oroxylum indicum* (L.) Kurz of Asian countries [23–24]. Accumulating studies have shown the beneficial biological effects of OA, which include antioxidant, antidiabetic, anticancer, antibacterial, anti-inflammatory and antiviral properties [25–30]. Herein, we selected Q[8] as a host molecule and investigated its host–guest interactions with OA, as well as its effect on the properties of OA. Our results provide an approach and theoretical basis for the development and utilization of oroxin A. Compared with the literature [31–32], it is found that although baicalein, oroxin B and oroxin A have the same aglycone, but the complex inclusion modes with Q[8] are different, It shows that the molecular size of the flavonoids and the length of the sugar chains have a greater impact on the assembly mode of supramolecular systems.

## Results and Discussion

### Host–guest interactions

**Figure 2:**
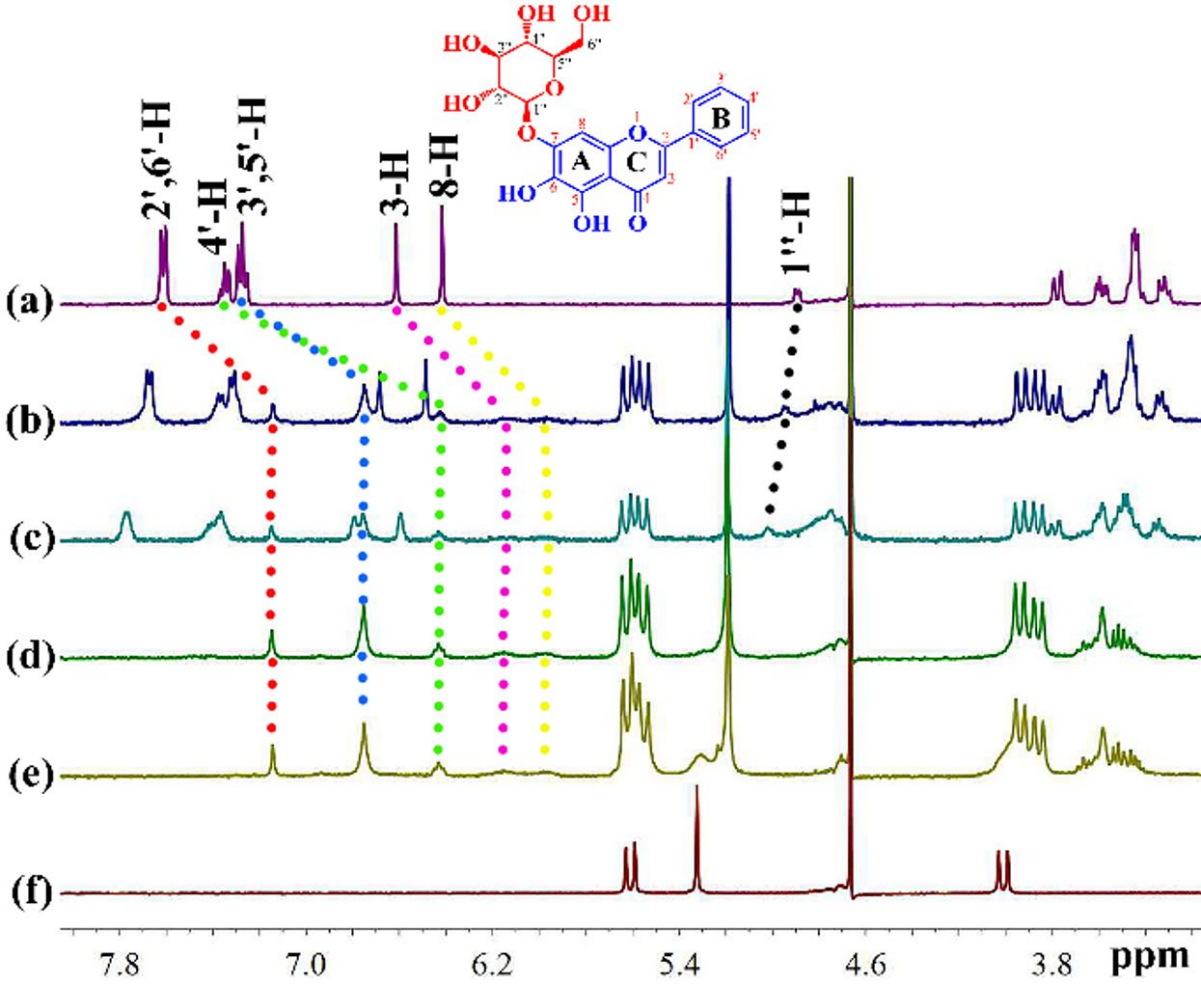
^1^H NMR titration of OA with Q[8] were performed in D_2_O containing 10% DMSO by volume, OA (500 μmol·L^−1^) upon the addition of different molar equivalents of Q[8]: (a) 0, ( b ) 0.35, (c) 0.44, (d) 1.03, (e) 1.60 and (f) neat Q[8].

The host–guest interaction can be effectively observed using ^1^H NMR spectroscopy, and the mode of action of the cucurbit[*n*]uril-guest can be inferred from the chemical shift changes of the guest proton resonance peaks. ^1^H NMR titration experiments were performed in D_2_O containing 10% DMSO by volume at room temperature. As shown in Figure 2 and Table 1, upon the addition of Q[8], some of the peaks of the protons of the OA aglycone shifted upfield, while the peaks due to the glycosidic proton shifted downfield. At the same time, the proton peaks of Q[8] were split, indicating that OA interacted with Q[8]. When the host–guest molar ratio was 1:1, all of the OA aglycone proton peaks moved upfield, indicating the entry into the cavity of Q[8]. The proton peaks of the glycosidic H and glucose were shifted downfield, indicating that they were located at the portal of Q[8]. However, when OA is present in excess, some of the proton peaks of the OA aglycon moved upfield, and some move downfield, indicating that in the case of an excess of OA, the port interactions and inclusion interactions of OA andQ[8] can exist simultaneously.

**Table 1:** Changes in the ^1^H NMR chemical shifts.

^1^H nucleus	Δδ/ppm

2',6'-H (cycle b)	0.47
4'-H (cycle b)	0.92
3',5'-H (cycle b)	0.52
3-H (cycle c)	0.46
8-H (cycle a)	0.45
1''-H (glycoside)	−0.13

**Figure 3:**
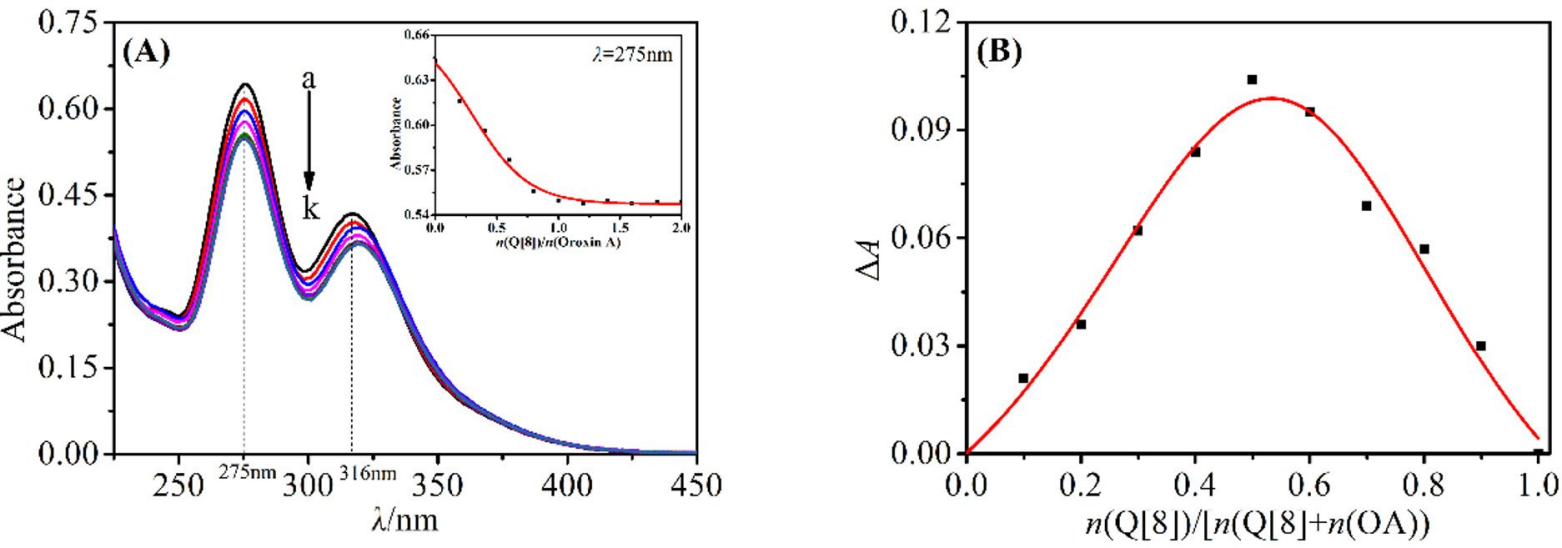
(A) The UV–vis absorption spectra recorded for OA in the presence of Q[8] (*c*(Q[8]), labeled a–k: 0, 0.2, 0.4, 0.6, ..., 2.0 × 10^–5^ mol·L^−1^) and (B) Job’s plot obtained for OA in the presence of Q[8].

To further determine the host–guest ratio of the inclusion complex formed by Q[8] and OA, their interaction was investigated using UV–visible absorption spectroscopy via a molar ratio method and Job's method. Figure 3A shows the UV–visible absorption spectra of the interaction between Q[8] and OA. It can be seen that the UV absorption of OA at 275 nm and 316 nm decreased significantly as the concentration of Q[8] was increased. When *n*(Q[8])/*n*(OA) = 1, there was a clear transition of the absorbance of the system. Upon further addition of Q[8], the absorption value of the system tended to be constant, indicating the formation of a 1:1 complex with a binding constant *K* = 1.299 × 10^7^ L·mol^−1^. The result of the Job’s plot also confirmed the combination of Q[8] and OA in a 1:1 mode (Figure 3B).

**Figure 4:**
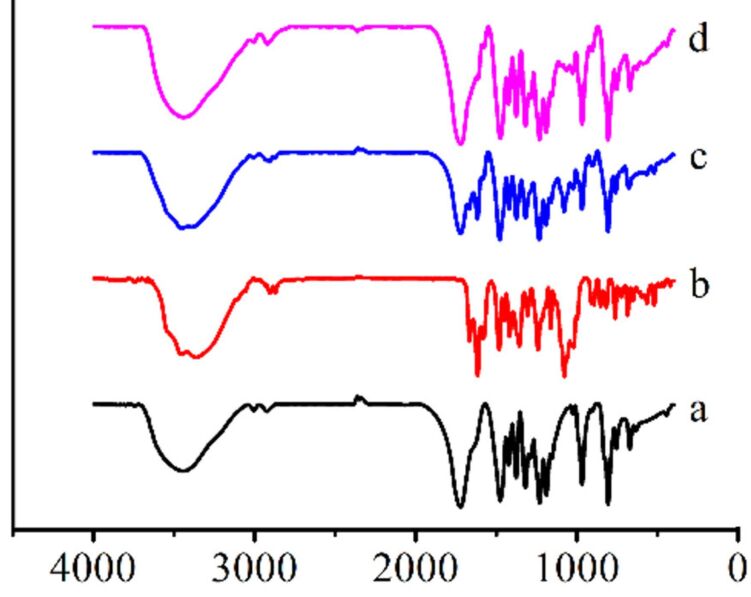
IR spectra recorded for (a) Q[8], (b) OA, (c) a physical mixture of Q[8] and OA, and (d) the OA@Q[8] inclusion complex.

Figure 4 shows the IR spectra recorded for Q[8] (a), OA (b), a physical mixture of Q[8] and OA (*n*(Q[8]):*n*(OA) = 1:1) (c) and the OA@Q[8] inclusion complex (d). Curve (c) contains characteristic peaks of curves (a) and (b) without interaction in the physical mixture. Comparing spectra (c) and (d), the peaks at 1617.41, 1482.23 and 1451.06 cm^−1^ due to stretching vibrations of the two benzene rings disappeared, and the peak at 1079.42 cm^−1^ due to C–O stretching vibrations was obviously weakened in the inclusion complex, which were caused by Q[8].

**Figure 5:**
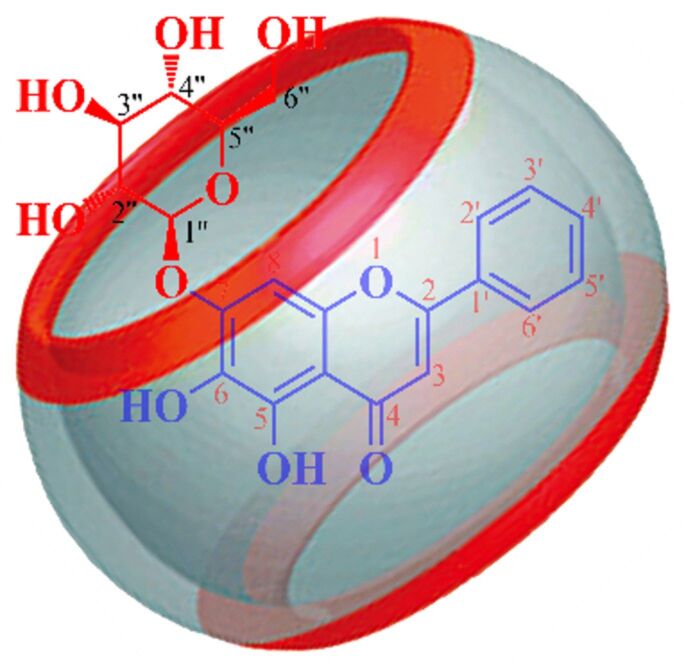
The possible interaction mode for OA@Q[8].

The mass spectrum of the OA@Q[8] inclusion complex featured the parent ion peak at *m/z* 1783.5716 [M + Na]^+^ (calcd. 1783.4983 [M + Na]^+^) (Supporting Information File 1, Figure S1), further supporting the formation of a 1:1 inclusion complex between OA and Q[8]. The possible host–guest mode is shown in Figure 5.

### The effect of OA on the properties of cucurbit[8]uril

#### Phase-solubility

**Figure 6:**
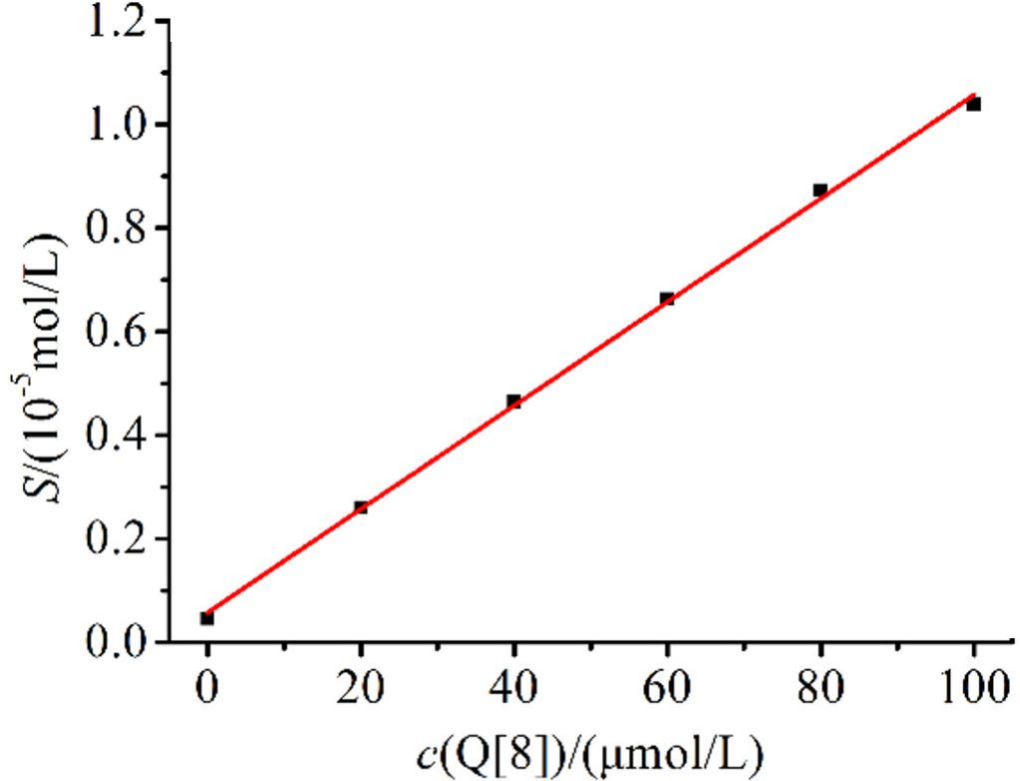
The phase-solubility graph obtained for OA in a Q[8] aqueous solution at λ = 275 nm.

Phase-solubility studies were conducted to investigate the solubility of OA in the presence of Q[8]. As can be seen from Figure 6, the solubility of OA in water is very poor (4.62 × 10^−6^ mol·L^−1^). The solubility of OA increased linearly in water with the addition of Q[8]. When the concentration of Q[8] was 1.0 × 10^−4^ mol·L^−1^, the solubility of OA was increased 22.47-fold. The solubility curve equation was *S* = 0.01*c* + 0.0575, *R*^2^ = 0.9986.

#### Antioxidant activity

**Figure 7:**
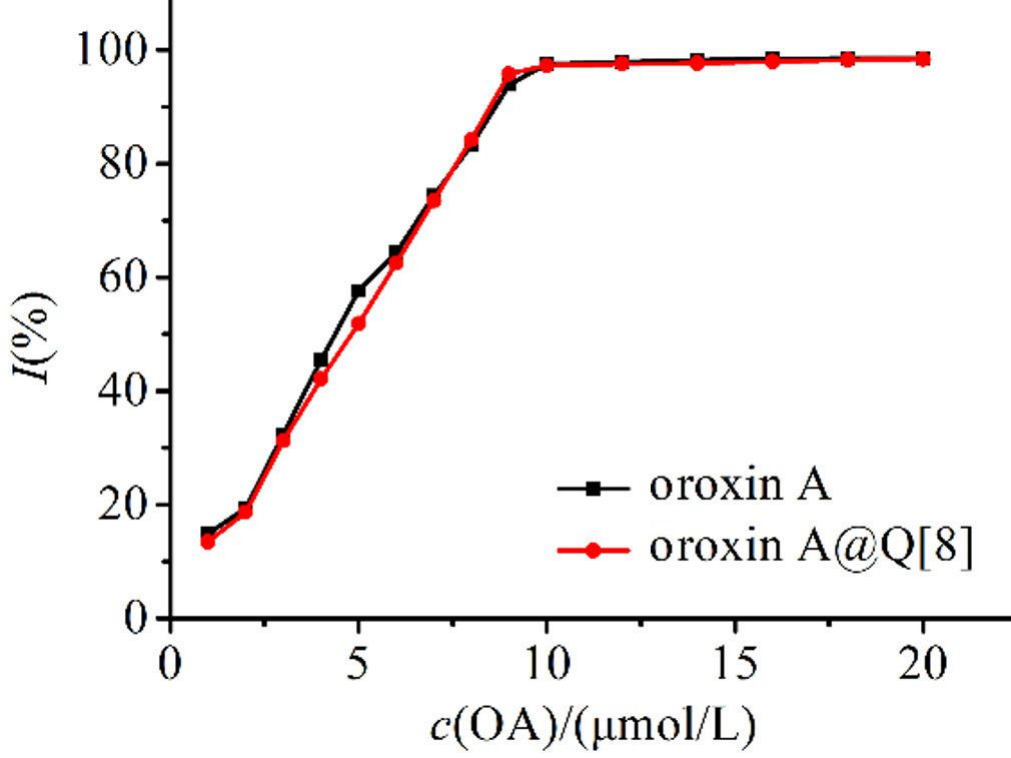
The clearance rate curve of ABTS^+•^ upon increasing the concentration of OA and the OA@Q[8] inclusion complex.

OA has strong antioxidant activity and effectively eliminates ABTS^+•^ radicals. If the antioxidant activity of OA was decreased significantly by the formation of the OA@Q[8] complex, the medicinal value of OA would be seriously affected. Figure 7 shows the results for OA and OA@Q[8] scavenging of ABTS^+•^ radicals in the range of 1–20 μmol·L^−1^. The IC_50_ values of OA and OA@Q[8] were 4.65 × 10^−6^ mol·L^−1^ and 4.80 × 10^−6^ mol·L^−1^, respectively, which indicates that Q[8] did not affect the antioxidant activity of OA.

#### Drug release in vitro

**Figure 8:**
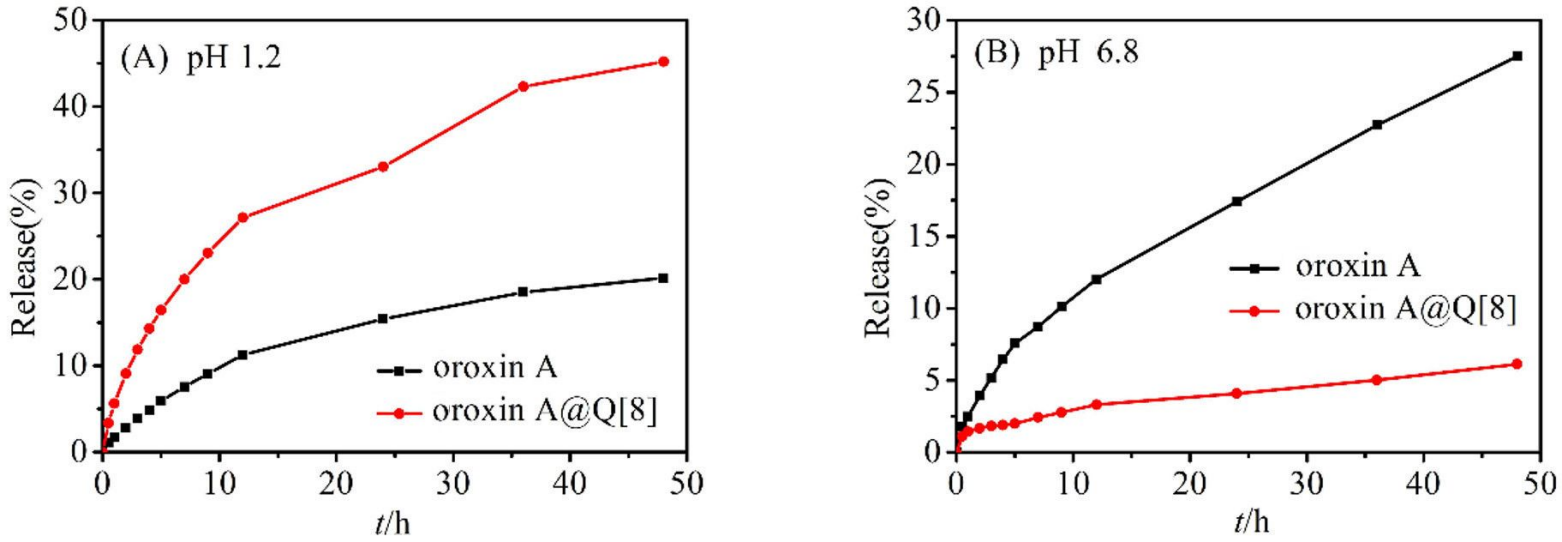
The release curves of OA and OA@Q[8].

Figure 8 shows the cumulative release of the OA and the OA@Q[8] inclusion complex in artificial gastric juice (pH 1.2) and artificial intestinal juice (pH 6.8). It can be seen from Figure 8A that release of OA@Q[8] inclusion compound in artificial gastric juice was much higher than that of OA after 12 h. The cumulative release of OA and OA@Q[8] reached 11.25% and 27.15%, respectively, after 12 h. After 48 h, Q[8] increased the measured cumulative release of OA in artificial gastric juice by 2.3-fold. In artificial intestinal fluid (Figure 8B), the release rate of OA was faster than that of OA@Q[8]. After 12 h, the cumulative release of OA was 12.02%, while there was only 3.31% release of OA@Q[8].

## Conclusion

In summary, the experimental results showed that OA and Q[8] formed a host–guest complex in a ratio of 1:1. The aglycone of OA entered the cavity of Q[8] and the glucose was located at the portal of Q[8], with a binding constant of 1.299 × 10^7^ L·mol^−1^. The solubility of oroxin A was increased 22.47-fold when the concentration of the added Q[8] was 1.0 × 10^−4^ mol·L^−1^. The results of the UV absorption spectrum analysis showed that Q[8] enhanced the cumulative release of OA in artificial gastric juice by 2.3-fold, but had no effect on its antioxidant activity.

## Supporting Information

File 1 Apparatus, materials and methods.
