# Supplementary material for: Host–guest interaction of cucurbit[8]uril with oroxin A and its effect on the properties of oroxin A
Source: Beilstein J Org Chem. 2020 Sep 22;16:2332–7. doi: 10.3762/bjoc.16.194 (PMC7522457; doi:10.3762/bjoc.16.194)
Supplement: File 1 — Apparatus, materials and methods. [file Beilstein_J_Org_Chem-16-2332-s001.pdf]

BEILSTEIN JOURNAL OF ORGANIC CHEMISTRY

Supporting Information

for

Host–guest interaction of cucurbit[8]uril with oroxin A and its effect on the properties of oroxin A

Zhishu Zeng, Jun Xie, Guangyan Luo, Zhu Tao and Qianjun Zhang

Beilstein J. Org. Chem. **2020,** *16,* 2332–2337. doi:10.3762/bjoc.16.194

Apparatus, materials and methods

License and Terms: This is a supporting information file under the terms of the Creative Commons Attribution License (https://creativecommons.org/licenses/by/4.0). Please note that the reuse, redistribution and reproduction in particular requires that the authors and source are credited.
The license is subject to the *Beilstein Journal of Organic Chemistry* terms and conditions: (https://www.beilstein-journals.org/bjoc)

Table of Contents

Material S2

Apparatus S2

Methods S2

- ^{1}H NMR and MS S2
- UV–vis measurements S3
- Synthesis of the inclusion complex S3
- IR spectroscopy S4
- Phase solubility method S4
- ABTS radical scavenging activity S4
- In vitro release studies S5

References S6

Material

Q[8] (purity ≥97%) was prepared in the Key Laboratory of Macrocyclic and Supramolecular Chemistry of Guizhou Province, China. 2,2'-Azino-bis(3-ethylbenzothiazoline-6-sulfonic acid) (ABTS), OA, $K_2S_2O_8$ and other reagents (purity ≥97%) were purchased from Sigma-Aldrich.

Apparatus

The used devices were: UV-2700 double beam UV–visible spectrophotometer; RE-52A rotary evaporator; VERTEX70 (Bruker, Germany) Fourier infrared spectrometer; FA2204N electronic balance; JNM-ECZ400s MHz nuclear magnetic resonance (NMR) spectrometer; SB-5200D ultrasonic instrument; SHY-2A Thermostatic oscillator; Agilent 6545 Q-TOF LC/MS.

Methods

^{1}H NMR and MS

A JEOL JNM-ECZ400s spectrometer was used to record the ^{1}H NMR spectra at 20 ℃. ^{1}H NMR titration experiments of OA with Q[8] were performed in D_2O containing 10% DMSO by volume.

ESI–TOF mass spectrometry of the OA-Q[8] inclusion complex was performed on an Agilent 6545 Q-TOF at room temperature. An aqueous solution of OA@Q[8] was prepared at a concentration of 1.00×10^{-4} mol·L^{-1}, then the solution was filtered and tested by MS.

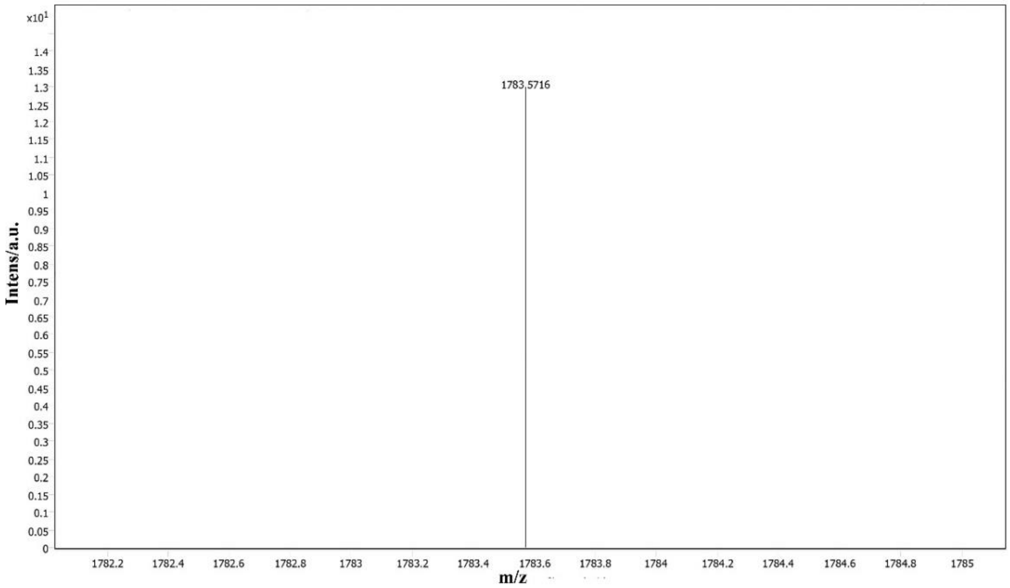

Figure S1: ESI–TOF mass spectrometry of the OA@Q[8] inclusion complex

UV–vis measurements

UV–visible spectra were recorded from samples in 1 cm quartz cells on UV-2700 double beam UV–visible spectrophotometer. The host and guests were dissolved in distilled water. UV-visible spectra were obtained at a OA concentration of 2 × 10^{-5} mol·L^{-1} and different Q[8] concentrations for the OA@Q[8] system. When the host–guest interaction ratio is 1:1, the binding constant *K* is calculated by the following formula:

$$\Delta A = \frac{\Delta\alpha([H]_0 + [G]_0 + 1/K) \pm \sqrt{\Delta\alpha^2([H]_0 + [G]_0 + 1/K)^2 - 4\Delta\alpha^2[H]_0[G]_0}}{2}$$

Where ΔA is the change in the absorbance of the Q[8] upon gradual addition of the OA, and Δα refers to the different constant of the free host and the interaction complex. The total concentrations of host and guest are expressed by $[H]_0$ and $[G]_0$, respectively [1].

Synthesis of the inclusion complex

The inclusion complex was prepared by the method of reference [2]. The requisite amount of Q[8] and OA were weighed according to the ratio

n(OA):n(Q[8]) = 1:1, dissolved in deionized water and the solutions were mixed and stirred for 1 h. The solvents were then evaporated to leave the OA@Q[8] inclusion complex (1:1).

IR spectroscopy

OA, Q[8], a mixture of OA and Q[8] (n(OA):n(Q[8]) = 1:1) and OA@Q[8] were weighed and mixed with dry KBr, respectively. The mixtures were pressed into a standard mold to prepare the required discs, and the infrared spectra measured over the wavenumber range of 4000–400 cm^{-1}.

Phase solubility method

Calibration curve for OA and OA@Q[8] standard solution: A series of different concentrations (from 5.0×10^{-6} $mol{\cdot}L^{-1}$ to 2.5×10^{-5} $mol{\cdot}L^{-1}$) of OA and OA@Q[8] solutions were measured at λ = 275nm and the standard calibration curve was obtained. The regression equation of OA is $A_1 = 30560c + 0.0148$, $R^2 = 0.9997$; and the regression equation of OA@Q[8] is $A_2 = 25900c + 0.0089$, $R^2 = 0.9999$.

The method for determination the phase solubility of OA [3]: 5.0 mg of OA were placed into 10 mL volumetric flasks and different volumes (0, 2, 4, 6, 8 and 10 mL) of a 1×10^{-4} $mol{\cdot}L^{-1}$ Q[8] solution added. Water was used to make up the remaining volume and the flasks were then subjected to ultrasonication for 60 min until a solid-liquid equilibrium was achieved. The absorbance was measured at λ = 275 nm after filtration through a 0.5 μm membrane filter. The content of OA was calculated according to the working curve.

ABTS radical scavenging activity

The antioxidant capacity was estimated based on radical scavenging activity according to the method described by Delgado-Andrade et al. [4]. The

$ABTS^{+•}$ stock solution was prepared by mixing 7.0 mmol·L^{-1} of 2,2-diazobis(3-ethylbenzothiazol-6-sulfonic acid) diammonium salt (ABTS, aqueous solution) with 2.45 mmol·L^{-1} of a potassium persulfate solution by equal volume, and placing it in a dark room for 12–16 h at room temperature. The stock solution was diluted with 5 mmol·L^{-1} phosphate-buffered saline (pH 7.4) to an absorbance of 0.70 ± 0.02 at λ = the 730 nm. After the addition of 100 μL of different concentrations of sample (OA and OA@Q[8]) to 10 mL of diluted $ABTS^{+•}$ solution, the absorbance was measured at 20 min to evaluate the effect of Q[8] on the antioxidant activity of OA. The percentage inhibition displayed by the test compounds was calculated using the following equation:

$$I(\%) = [(A_{control} - A_{sample})/A\ control] \times 100\%$$

Where $A_{control}$ is the absorbance of the control reaction solution (containing all the reagents except the test compound) and A_{sample} is the absorbance of the reaction solution in the presence of the test compound. The half-inhibition concentration (IC_{50}) was calculated using the clearance rate curve.

In vitro release studies

The dialysis bag method [5] was used to investigate the in vitro release behavior of the OA@Q[8] inclusion complex. 3.0 mg of OA and 6.8 mg of the OA@Q[8] inclusion compound (with the same mass of OA) were accurately weighed and placed in a dialysis bag (molecular weight cut-off: 500). The dialysis bag was sealed and placed in 100 mL of artificial gastric juice (pH = 1.2 hydrochloric acid solution) or artificial intestinal fluid (pH = 6.8 phosphate buffer solution) and shaken in a water bath at 37 °C. At appropriate time intervals, aliquots (3 mL) of the sample solution were removed (while replenishing the same volume of fresh release medium 3 mL) and the absorbance of the samples was measured at 275 nm. The release mass was obtained according to the absorbance of OA, and the cumulative release of OA was calculated by the following formula.

$$R(\%) = \frac{m_{release}}{M_{total}} \times 100\%$$

Where *R*(%) is the cumulative release; $m_{release}$ is the mass released by OA; M_{total} is the total mass of OA.

References

1. Thordarson, P. *Chem. Soc. Rev*. **2011**, 40, 1305-1323.
2. Xie, J.; Zeng, Z.; Tao, Z.; Zhang, Q. *Chem. Res. Chinese U.* **2020, DOI:** 10.1007/s40242-020-0005-6 (prepublish).
3. Zhang, X.; Xie, J.; Xu, Z.; Tao, Z.; Zhang, Q. *Beilstein J. Org. Chem.* **2020**, 16, 71–77.
4. Delgado-Andrade, C.; Rufián-Henares, J.; Morales, F. J. *J. Agr. Food. Chem.* **2005**, 53, 7832-7836.
5. Fontana, M. C.; Coradini, K.; Pohlmann, A. R.; Guterres, S. S.; Beck, R. C. R. *J. Nanosci. Nanotechno.* **2010**, 10, 3091-3099.
